# Study of the Drying Kinetics on the Nutritional and Technological Quality of Dried Pasta Enriched With Coffee Pulp Flour

**DOI:** 10.1111/1750-3841.70673

**Published:** 2025-11-07

**Authors:** Betsy Gois Santos, Giovana Toscano Ancillotti, Carolina Hage de Figueiredo, Mariana Aranda Rodrigues, Vanessa Naciuk Castelo‐Branco, Thais Matsue Uekane

**Affiliations:** ^1^ Faculdade de Farmácia, Departamento de Bromatologia Universidade Federal Fluminense Niterói Rio de Janeiro Brazil

**Keywords:** by‐products, dietary fiber, food enrichment, food waste valorization, sustainability

## Abstract

This study investigated the drying kinetics of dried pasta fortified with coffee pulp flour (CPF), assessing its potential as a functional ingredient relative to commercial whole wheat flour (WWF) and the subsequent impact on final product quality. CPF demonstrated a five‐fold higher dietary fiber content (50%) and exhibited superior technological properties, including water absorption (6 g water/g) and solubility (26%), thereby indicating its promise for food product development. Pastas were formulated with partial replacement of refined wheat flour (WF) by CPF or WWF at concentrations corresponding to 10%, 20%, and 25% of the recommended daily intake of fiber. Drying was conducted at 45°C, 55°C, and 65°C. The drying kinetics were described by the Page, Logarithmic, and Henderson and Pabis models, with the Page model providing the best fit (adjusted R^2^ ≥ 0.98; standard error < 1.0 and < RMSE values). A drying temperature of 65°C proved most effective for pasta processing, leading to accelerated drying and reduced cooking time (8–10 min). Notably, even with lower CPF incorporation, the resulting pasta achieved fiber contents comparable to or exceeding those with WWF. Furthermore, these pastas exhibited increased total phenolic content, elevated levels of bioactive compounds such as caffeine (40–109 mg/80 g), trigonelline (24–56 mg/80 g), and 5‐caffeoylquinic acid (5–7 mg/80 g), and enhanced antioxidant capacity, thus confirming the efficacy of enrichment. This research underscores the technological and nutritional viability of CPF as a functional and sustainable ingredient, thereby contributing to the valorization of agro‐industrial by‐products and promoting increased dietary fiber intake.

**Practical Applications:**

This research shows that it is possible to produce pasta rich in dietary fiber and with bioactive potential using coffee pulp flour, a by‐product of the coffee industry, as a food ingredient. In addition to improving the nutritional value of pasta, this innovation can reduce agricultural waste and offer new functional options for the food industry.

## 1 Introduction

Pasta products are widely consumed worldwide, traditionally made with wheat flour; their consumption is associated with practicality in preparation, culinary versatility, low cost, and good sensory acceptance (Dziki 2021). According to data from the International Pasta Organization (IPO), Brazil is the third largest producer and consumer of pasta products in the world, with an average consumption of approximately 6 kg per person/year, which reinforces the relevance of nutritional enrichment strategies for this food matrix.

Coffee, genus Coffea, is the second most traded product in the world. Its post‐harvest wet processing consists of the removal of the pulp and mucilage from the beans through mechanical action, followed or not by fermentation in tanks. This process gives rise to a by‐product called “coffee pulp,” which represents between 30% and 50% of the total fruit weight. This coffee pulp is generally discarded as waste or eventually used as fertilizer. Additionally, this coffee pulp has great potential for use in foods, due to the high content of fibers, minerals, bioactive compounds, and antioxidants, and its valorization as a food ingredient usable by the food industry is of interest (Klingel et al. 2020).

It is known that adequate consumption of dietary fibers is fundamental for health, contributing to the improvement of digestive function, glycemic and cholesterol control, regulation of body weight, and prevention of non‐communicable chronic diseases (Tolve et al. 2020; Liu et al. 2019). However, studies indicate that global fiber intake is below the level recommended by the WHO of 25 g/day (Liu et al. 2019). Thus, there has been growing interest in the development of foods enriched with fibers aiming to meet this demand.

Although pasta is a widely consumed food product, versions produced mostly with refined wheat flour tend to present a low fiber content. Because of this, the enrichment of this matrix with fibrous and bioactive ingredients has emerged as a strategy to improve the nutritional profile without mischaracterizing the product (Aranibar et al. 2018). In this context, coffee pulp flour stands out for combining a high content of fiber and bioactive compounds with wide availability as an agro‐industrial by‐product, especially in producing countries such as Brazil, aligning with valorization (upcycling) initiatives.

Beyond the nutritional aspects, the incorporation of coffee pulp flour responds to an agenda of circular economy and territorial development of the coffee chain by adding value to an abundant residue, reducing organic disposal and environmental costs, and being in accordance with sustainable development goals (SDGs)—Responsible Consumption and Production (Tsigkou et al. 2025). Furthermore, it makes it possible to diversify producers’ sources of income with new marketing routes throughout the year, consolidating technically viable upcycling strategies through a widely accepted matrix (pasta) to expand access to foods with better quality density without requiring significant changes in eating habits.

Accordingly, this study developed a dry pasta enriched with coffee pulp flour and evaluated its drying kinetics and nutritional quality, adopting whole wheat flour as a technological benchmark, to compare process performance, and study a sustainable alternative for enriching products with fibers, and the impacts on the quality of the final product.

## 2 Materials and methods

### 2.1 Raw Material

The coffee pulp was obtained by donation from a producer in the mountainous region of Rio de Janeiro state (São José do Vale do Rio Preto, 22° 09′ 05″ S, 42° 55′ 28″ W, 615 m altitude) from the 2024 harvest. Other ingredients used in the pasta production were purchased from local supermarkets in the city of Niterói (RJ, Brazil). Coffee pulp was dried in a ventilated oven at 55°C for approximately 48 hours, until it reached ≤12% moisture content. Dried coffee pulp was stored in hermetic grain storage bags (GrainPro). The coffee pulp flour (CPF) was obtained by grinding the dried pulp in a blender (Philco, Manaus, Brazil), followed by milling in a household grinder (Mr. Coffee‐IDS 57, China), and sieving through a 250 µm (60 mesh) tamis. The CPF was stored in sealed polyethylene and polyamide bags and kept in desiccators at room temperature (25 ± 2°C) until use.

### 2.2 Solvents and Chemical Reagents

Methanol (MeOH), acetic acid, hydrochloric acid (HCl), were all HPLC grade, ethanol P.A. and acetone P.A. were purchased from Êxodo Científica (São Paulo, Brazil). Other reagents used were Folin–Ciocalteu reagent (2N, ASC Científica), FRAP reagent (Neon Química), TPTZ (2,4,6‐Tris(2‐pyridyl)‐s‐triazine—Neon Química), DPPH (2,2‐diphenyl‐1‐picrylhydrazyl—Sigma‐Aldrich D9132), Trolox (Sigma‐Aldrich 648471), Caffeine (Sigma‐Aldrich C0750, >99%), Trigonelline (Sigma‐Aldrich T5509, >99%), and 5‐CQA (Sigma‐Aldrich C3878, >95%).

### 2.3 Proximate Composition and Technological Properties of the Flours

Proximate composition analyses of coffee pulp flour (CPF), whole wheat flour (WWF), and refined wheat flour (WF) were performed in triplicate, according to the Adolfo Lutz Institute: moisture (012/IV), total ash (018/IV), protein (036/IV) using a conversion factor of 5.75 for CPF and 5.83 for WWF and WF, gluten (418/IV), lipids (032/IV), and carbohydrates (Nifext method) (IAL 2008). Soluble fiber (SF), insoluble fiber (IF), and total fiber (TF) were determined using the AOAC 985.29 enzymatic‐gravimetric method (AOAC 2005). All results were expressed on a dry basis.

Technological properties assessed included water absorption index (WAI), oil absorption index (OAI), water solubility index (WSI), and swelling volume (SV), all in triplicate following the methods described by Robertson et al. (2000).

### 2.4 Dried Pasta Formulation

Based on the results of the average dietary fiber content in the flours, formulations were calculated with partial replacement of refined wheat flour to meet 10%, 20%, and 25% of the Dietary Reference Intakes (DRI) value. According to the composition and labeling criteria established by Brazilian legislation (IN n° 75/2020) (Brazil 2020), for a food product to bear health claims related to fiber content, it must meet the minimum values based on the DRI. Considering the World Health Organization's recommendation of a daily intake of 25 g of fiber, a food is considered a “source of fiber” when it provides at least 2.5 g per serving (10% of the DRI), is classified as “high in fiber” when it contains at least 5 g per serving (20% of the DRI), and may be labeled as “fiber‐enriched” if it provides at least 6.25 g per serving (25% of the DRI).

These formulations were named as follows: WWF 10 (partial replacement of WF by whole wheat flour (WWF), providing 10% of the DRI), CPF 10 (partial replacement of WF by coffee pulp flour (CPF), providing 10% of the DRI), WWF 20 (partial replacement of WF by WWF, providing 20% of the DRI), CPF 20 (partial replacement of WF by CPF, providing 20% of the DRI), WWF 25 (partial replacement of WF by WWF, providing 25% of the DRI), and CPF 25 (partial replacement of WF by CPF providing 25% of the DRI). The formulations are detailed in Table 1.

**TABLE 1:** Formulations and proportion of ingredients used in the production of dried pasta with partial replacement of refined wheat flour by whole wheat flour or coffee pulp flour.

	10% DRI		20% DRI		25% DRI	
Ingredients	WWF 10	CPF 10	WWF 20	CPF 20	WWF 25	CPF 25
**White flour (g)**	160	192	120	184	88	177,6
**Whole wheat flour (g)**	40	—	80	—	112	—
**Coffee pulp flour (g)**	—	8	—	16	—	22,4
^1^ **Egg (g)**	24	24	24	24	24	24
**Water (mL)**	10	10	15	15	18	18

^1^ Whole dehydrated eggs were used. WWF 10 = partial replacement of WF by WWF providing ‐10% of the DRI for fiber; CPF 10 = partial replacement of WF by CPF ‐providing 10% of the DRI for fiber; WWF 20 = partial replacement of WF by WWF ‐providing 20% of the DRI for fiber; CPF 20 = partial replacement of WF by CPF ‐providing 20% of the DRI for fiber; WWF 25 = partial replacement of WF by WWF ‐providing 25% of the DRI for fiber; CPF 25 = partial replacement of WF by CPF ‐providing 25% of the DRI for fiber.

The pasta was produced using an automatic pasta machine (Emeril Lagasse, New Jersey, USA), which performs mixing, extrusion, and shaping steps (into *penne* format) automatically. After this process, the pasta was manually cut to a predefined length (4 cm) using a millimeter ruler and then placed in a ventilated oven for drying. The objective of this study was to evaluate the influence of coffee pulp flour incorporation on the drying kinetics of dry pasta, compared to a commercial whole wheat flour. The control formulation maintained the same blend ratio of refined wheat flour and whole wheat flour used as the base for the enriched pastas, ensuring equivalence in the final fiber content. An additional group, consisting of dried pasta 100% White Flour pasta (WF 100%), was included in the Supplementary Material.

### 2.5 Drying Kinetics

Fresh pasta samples were arranged on perforated trays (50 × 25 cm, 1 cm in height) in rows with 1 cm spacing, and placed in a ventilated oven (Solab SL‐102, São Paulo, Brazil) with hot air circulation (1.5 m/s). at different drying temperatures (45°C, 55°C, and 65°C). Subsequently stored in sealed polyethylene and polyamide bags, maintained at 25°C ± 2°C until analysis.

Moisture content was recorded at 15 min intervals during the first hour, every 30 min during the second and third hours, and every 60 min until a total of 6 h was reached. Trays were manually rotated 180° at each sampling point to minimize positional effects during drying. Moisture content was determined using an infrared moisture analyzer (model ID 200, Marte Científica, Minas Gerais, Brazil). To construct the drying curves as a function of time, the mathematical models of Page (1949), Logarithmic (1973), and Henderson and Pabis (1961) were applied by replacing the experimental moisture ratio (MR) with the measured moisture values over time. The experimental MR was determined for each temperature according to Equation 1.

(1) MR=Mt/M0

where, Mt is the moisture content (%) at any given time, and M0 is the initial moisture content (%).

Mathematical models of Page (1949), Logarithmic (1973), and Henderson and Pabis (1961) were fitted to the experimental data according to Equation 2, Equation 3, and Equation 4, respectively.

(2) $$\mathrm{MR}=\exp\;\left(-k.t^{n}\right)\;$$

(3) $$\mathrm{MR}=a.\exp\;\left(-k.t\right)+c$$

(4) $$\mathrm{MR}=a.\exp\left(-k.t\right)$$

where, k is the drying constant, *a*, *c*, and *n* are the coefficients specific to each model, and t is the drying time.

After determining the coefficients based on the experimental data obtained, the equations were rearranged by isolating the time parameter (t) to construct the drying curve.

### 2.6 Optimal Cooking Time

The optimal cooking time was determined according to Ermişer and Yalçın (2021) a sample of 10.0 g of pasta was cooked in 500 mL of boiling distilled water. At predetermined time intervals (3, 6, 8, 9, 10, 11, 12 min), the samples were compressed between two glass plates until the white core at the center of the pasta disappeared. At that point, the pasta was considered fully cooked.

### 2.7 Physicochemical Characterization

The physicochemical analyses were performed on the pasta samples dried at the temperature that showed the best performance based on drying kinetics and cooking quality. The following parameters were evaluated: moisture (413/IV), alcohol‐soluble acidity (ATT) (415/IV), total ash (018/IV), and dry gluten (418/IV), according to the methods of the Adolfo Lutz Institute (IAL 2008), all results were expressed on a dry basis. Color was measured using a portable colorimeter (ColorMeter Pro, Engecolor, Guangdong, China) and expressed as L*, a*, and b* values (CIELab system). Soluble, insoluble, and total dietary fiber contents were determined as described in AOAC (2005).

### 2.8 Bioactive Compounds and Antioxidant Activity

The extraction of bioactive compounds—caffeine, trigonelline, and 5‐CQA—was performed using 250 mg of ground dried pasta and 25 mL of acidified aqueous methanol (80%, v/v; 1% HCl, v/v), with agitation for 2 h followed by centrifugation at 3,000 rpm for 15 min. The supernatant was filtered through a Whatman n. 1 filter paper and sequentially a 0.45 µm PTFE membrane and stored at −20°C until analysis, adapted from Tolve et al. (2020). The contents of bioactive compounds were identified and quantified by high‐performance liquid chromatography with diode‐array detection (HPLC‐DAD) following the methods described by Vidal et al. (2022). The chromatographic system used was a Shimadzu (Kyoto, Japan) equipped with an LC20AT quaternary pump, SPD‐M20A DAD detector, DGU‐20A5R degasser, SIL‐20AC automatic injector, and CMB20A control module, operated via LabSolutions LC software. Chromatographic conditions were as follows: injection volume of 20 µL, flow rate of 1 mL/min, column oven at 30°C, and detection wavelengths set at 272 nm for caffeine and trigonelline, and 328 nm for 5‐CQA. Separation was achieved using a reversed‐phase C18 analytical column (4.6 mm × 250 mm, 5 µm, Kromasil 100, Bohus, Sweden). The mobile phases consisted of ultrapure water (A) and methanol (B), both acidified with 0.1% (v/v) acetic acid, under the following gradient conditions: 15% B (0–5 min), 15%–80% B (5–30 min), 80%–100% B (30–31 min), 100% B (31–35 min), and 100%–15% B (35–50 min). Calibration curves were prepared in the ranges of 1.0–50.0 µg/mL for caffeine and trigonelline, and 0.10–80.0 µg/mL for 5‐CQA, using ultrapure water. All analyses were performed in triplicate.

Total phenolic compounds were determined by the Folin–Ciocalteu method, as described by Singleton and Rossi (1965). Antioxidant activity was assessed using the ferric reducing antioxidant power (FRAP) assay and the free radical scavenging method (DPPH), according to Aranibar et al. (2018). Results were expressed as mg GAE of pasta (total phenolics), µmol Fe^2^⁺ equivalents (FRAP), and µmol Trolox equivalents (DPPH).

### 2.9 Statistical Analyses

The obtained data were subjected to normality assessment using the Shapiro–Wilk test. Descriptive statistics (mean and standard deviation) were then calculated for all evaluated parameters. Differences among treatments were analyzed by one‐way analysis of variance (ANOVA), and when a significant difference was found (*p* < 0.05), Tukey's test was applied for multiple comparisons. Statistical analyses were conducted using Jamovi software, version 2.3 (Sydney, Australia, 2022), and GraphPad Prism, version 8.0 (San Diego, USA, 2018); the latter was used for fitting the models applied to the drying kinetics.

## 3 Results and Discussion

### 3.1 Proximate Composition and Technological Properties of the Flours

Results for coffee pulp flour (CPF), whole wheat flour (WWF), and refined wheat flour (WF) used in the dry pasta formulations are presented in Table 2.

**TABLE 2:** Proximate composition and technological properties results of the coffee pulp flour (CPF), whole wheat flour (WWF), and refined wheat flour (WF).

	*Proximate composition*		
Parameters (%)	CPF	WWF	WF
**Moisture**	8.59 ± 0.30^a^	9.90 ± 0.11^b^	12.4 ± 0.15^c^
**Ash**	9.93 ± 0.05^a^	1.54 ± 0.02^b^	0.74 ± 0.05^c^
**Lipid**	2.81 ± 0.20^a^	2.54 ± 0.19^a^	2.72 ± 0.28^a^
**Protein**	12.3 ± 0.16^a^	13.4 ±0.40^b^	11.5 ± 0.14^c^
**Soluble fiber**	8.04 ± 1.77^a^	1.26 ± 0.81^b^	1.92 ± 0.03^b^
**Insoluble fiber**	42.6 ± 5.46^a^	10.0 ± 0.10^b^	1.77 ± 0.23^c^
**^1^ Total fiber**	50.7 ± 5.17^a^	11.3 ± 0.91^b^	3.70 ± 0.17^c^
**Carbohydrate**	24.6 ± 0.16^a^	71.4 ± 0.60^b^	81.3 ± 0.16^c^
**Dry gluten**	—	9.29 ± 0.10^a^	11.5 ± 1.10^a^
	***Technological properties***		
**WAI (g água/g)**	6.44 ± 0.22^a^	3.30 ± 0.50^b^	1.83 ± 0.03^c^
**OAI (g óleo/g)**	2.65 ± 0.15^a^	2.54 ± 0.09^a^	1.80 ± 0.35^b^
**WSI (%)**	26.0 ± 0.28^a^	10.3 ± 1.90^b^	2.26 ± 0.37^c^
**SV (mL/g^−1^)**	11.8 ± 0.76^a^	10.3 ± 0.41^a^	9.66 ± 0.06^ab^

The results were expressed as mean ± standard deviation of triplicate analyses. Means followed by the same letter in the same row do not differ significantly according to ANOVA, followed by Tukey's test (p < 0.05). ^1^Total fiber was calculated as the sum of soluble and insoluble fiber fractions. WAI = water absorption index; OAI = oil absorption index; WSI = water solubility index; SV = swelling volume.

The moisture content of CPF was lower compared to WWF and WF, showing a significant difference (*p* < 0.05) among the three flours. This result was expected, as CPF underwent a more intensive pre‐drying process aimed at improving processing and preserving the raw material. Additionally, the fibrous nature of CPF may contribute to reducing the residual water content compared to wheat flour. This is an advantageous characteristic, as a lower moisture content contributes to greater microbiological stability and shelf life of the products formulated with this ingredient. When comparing 39 types of refined and whole wheat flours, Bodor et al. (2024) found that the lowest moisture content was observed in whole wheat flours (∼ 9%), which is consistent with the findings of this study when comparing WF and WWF. This could support the idea that the fiber content in the matrix may facilitate the reduction of moisture content.

CPF showed a significantly higher total ash content (*p* < 0.05) compared to wheat flours. This can be attributed to CPF's specific composition, which is rich in minerals derived from the plant's husk and cellulose, containing high concentrations of potassium, calcium, magnesium, and phosphorus (Iriondo‐DeHond et al. 2020). CPF stands out in this regard when compared to other food sources commonly used in the food industry, such as semolina (0.68%), quinoa (2.17%), and oats (1.97%) (Lamacchia et al. 2010).

There were no significant differences in lipid content among the flours, with average values around 3%, suggesting that the addition of these flours is unlikely to alter the lipid content of the formulations.

The protein content of WWF was higher than that of CPF and WF, with significant differences observed (*p* < 0.05). However, it is important to highlight that CPF does not contain gluten in its composition. Gluten is a complex protein formed by the gliadin and glutenin fractions, responsible for the formation of the protein network in baked products (Rizzo and Baroni 2018). The absence of gluten in CPF may represent both an advantage and a limitation, depending on the intended application. This characteristic makes it a viable alternative ingredient for the development of foods aimed at individuals with gluten intolerance or celiac disease, although it does not possess the network‐forming properties that gluten provides (Rizzo and Baroni 2018).

The main difference among the flours was observed in terms of dietary fiber content. CPF showed a total fiber content approximately five times higher than that of WWF and about ten times higher than that of WF. The results confirm that CPF is a source of fiber and is comparable to the fiber content of green banana flour (56%), which has been studied as a functional ingredient (Çetin‐Babaoğlu et al. 2024). Therefore, CPF could be considered a viable food ingredient option for enriching products with fiber when compared to traditionally used flours, such as wheat flours, even whole wheat. The use of flours rich in dietary fiber in food production could promote fiber intake and potentially help meet the recommended daily requirements (Tolve et al. 2020).

Given the observed characteristics, it is important to assess the impact of CPF addition on the properties of the final products.

The assessment of the technological properties of the flours showed that the water absorption index (WAI) was significantly higher for CPF, followed by whole wheat flour (WWF) and refined wheat flour (WF). This behavior could be attributed to the content of insoluble fibers, which influence the amount of water that remains bound to the hydrated fiber after the application of an external force. This is desirable depending on the type of product, as it can improve texture, yield, and moisture content in formulations (Leão et al. 2017). Sandhu et al. (2017) reported a WAI of 1.67–1.95 g water/g for flours from different oat cultivars, considered a matrix with high hygroscopic capacity, values lower than those of CPF, suggesting that CPF could have a good water‐holding capacity.

Regarding the Oil Absorption Index (OAI), CPF and WWF showed similar values (*p* > 0.05) and were higher than that of refined flour. Leão et al. (2017) explained that the composition of the food matrix, including proteins and fibers, influences the oil‐holding capacity due to the presence of hydrophilic and hydrophobic functional groups. The authors found an OAI of 1.23–1.35 g oil/g for flours from the epicarp and mesocarp of pequi, values lower than those observed for the flours in this study. This property is considered interesting for applications in products that require higher flavor retention (Leão et al. 2017).

The Water Solubility Index (WSI) was significantly higher for CPF compared to wheat flours (*p* < 0.05). According to Leão et al. (2017), this characteristic can positively influence the texture and gel‐forming ability in food products. The authors reported WSI values ranging from 16.7% to 19.84% for flours obtained from pequi by‐products, values that were higher than those of wheat flours but lower than that of CPF, indicating a greater solubility in aqueous medium. Water solubility is an important property, as it affects the successful incorporation of fiber‐rich ingredients into food products (Benitez et al. 2019).

Benitez et al. (2019) reported a swelling volume (SV) of 14.0 mL/g^−1^ for coffee parchment flakes, which was higher than that of the CPF. This result suggests that CPF has an SV like that of wheat flours, a characteristic that may contribute to improving the structure and softness of final products (Leão et al. 2017).

These findings highlight the potential of CPF compared to wheat flours, suggesting its viability for use by the food industry in product enrichment, while also promoting the sustainable development of the coffee production chain and the appropriate management of agro‐industrial residues.

### 3.2 Drying Kinetics of Pasta

The parameters obtained from the Page, Logarithmic, and Henderson and Pabis mathematical models were applied to the drying curves of the pasta. The adjusted models were compared by the adjusted coefficient of determination (R^2^_adj_), the standard error of the regression (S), and the root mean squared error (RMSE) (Table 3).

**TABLE 3:** Parameters of the Page, Logarithmic, and Henderson and Pabis models for the drying curves of the dried pastas.

Model	Formulation	Temperature (°C)	k	n	a	c	R^2^_adj_	S	RMSE
**Page**	WWF 10	45	0.00671	0.8166	32.1	—	0.9967	0.3214	0.2689
		55	0.00702	0.8589	31.9	—	0.9975	0.3347	0.2800
		65	0.01086	0.8177	32.8	—	0.9863	0.9597	0.8029
	CPF 10	45	0.00670	0.8254	31.8	—	0.9975	0.2873	0.2404
		55	0.00587	0.8961	31.9	—	0.9984	0.2712	0.2269
		65	0.01086	0.8177	32.6	—	0.9915	0.6872	0.5750
	WWF 20	45	0.00805	0.7916	33.2	—	0.9924	0.5111	0.4276
		55	0.00654	0.8883	32.7	—	0.9968	0.4096	0.3427
		65	0.01108	0.8345	33.9	—	0.9984	0.3256	0.2724
	CPF 20	45	0.00705	0.8361	32.8	—	0.9970	0.3464	0.2898
		55	0.00945	0.7942	33.5	—	0.9987	0.2372	0.1985
		65	0.01015	0.8415	33.5	—	0.9953	0.5498	0.4600
	WWF 25	45	0.00686	0.8409	33.3	—	0.9958	0.4112	0.3440
		55	0.00957	0.8334	33.1	—	0.9945	0.5563	0.4654
		65	0.01428	0.7925	33.9	—	0.9944	0.6146	0.5142
	CPF 25	45	0.00833	0.8027	35.1	—	0.9892	0.6845	0.5727
		55	0.00934	0.8116	33.8	—	0.9921	0.6216	0.5201
		65	0.01502	0.7775	35.5	—	0.9966	0.4916	0.4113
**Logarithmic**	WWF 10	45	0.00448	—	21.7	10.1	0.9981	0.2416	0.2021
		55	0.00442	—	26.1	5.35	0.9951	0.4639	0.3881
		65	0.00768	—	26.6	5.74	0.9920	0.7345	0.6145
	CPF 10	45	0.00435	—	22.5	8.89	0.9968	0.3220	0.2694
		55	0.00401	—	28.1	3.43	0.9964	0.4116	0.3444
		65	0.00630	—	25.6	6.48	0.9939	0.5832	0.4879
	WWF 20	45	0.00514	—	21.6	11.2	0.9976	0.2857	0.2390
		55	0.00494	—	27.1	5.47	0.9988	0.2456	0.2055
		65	0.00630	—	28.4	4.97	0.9971	0.4425	0.3702
	CPF 20	45	0.00419	—	25.4	6.85	0.9932	0.5207	0.4356
		55	0.00504	—	24.5	8.35	0.9946	0.4825	0.4037
		65	0.00608	—	27.7	5.29	0.9960	0.5035	0.4213
	WWF 25	45	0.00457	—	24.8	8.14	0.9970	0.3528	0.2952
		55	0.00588	—	26.3	6.44	0.9968	0.4223	0.3533
		65	0.00724	—	27.1	6.15	0.9950	0.5830	0.4878
	CPF 25	45	0.00757	—	21.4	7.46	0.8991	2.1010	1.7578
		55	0.00592	—	24.6	8.92	0.9993	0.9710	0.8124
		65	0.00724	—	27.8	6.89	0.9963	0.5147	0.4306
**Henderson and Pabis**	WWF 10	45	0.00236	—	31.1	—	0.9844	0.6935	0.6203
		55	0.00320	—	31.0	—	0.9913	0.6205	0.5550
		65	0.00508	—	31.3	—	0.9752	1.2880	1.1520
	CPF 10	45	0.00249	—	30.8	—	0.9867	0.6564	0.5871
		55	0.00330	—	31.3	—	0.9954	0.4672	0.4179
		65	0.00404	—	31.2	—	0.9803	1.0480	0.9374
	WWF 20	45	0.00247	—	31.9	—	0.9759	0.9088	0.8129
		55	0.00354	—	32.0	—	0.9929	0.6071	0.5430
		65	0.00457	—	32.6	—	0.9892	0.8562	0.7658
	CPF 20	45	0.00280	—	31.8	—	0.9884	0.6807	0.6088
		55	0.00298	—	32.0	—	0.9832	0.8552	0.7649
		65	0.00433	—	32.3	—	0.9875	0.8930	0.7987
	WWF 25	45	0.00280	—	32.3	—	0.9875	0.7168	0.6411
		55	0.00386	—	31.9	—	0.9852	0.9098	0.8137
		65	0.00473	—	32.3	—	0.9790	1.1910	1.0653
	CPF 25	45	0.00274	—	33.8	—	0.9749	1.0470	0.9365
		55	0.00329	—	32.5	—	0.9788	1.0210	0.9132
		65	0.00457	—	33.6	—	0.9780	1.2510	1.1189

k = drying constant; a, c, and n = model coefficients (dimensionless); R^2^adj = adjusted coefficient of determination; S = standard error of regression; **RMSE =** Root Mean Squared Error. WWF 10 = partial replacement of WF by WWF providing ‐10% of the DRI for fiber; CPF 10 = partial replacement of WF by CPF ‐providing 10% of the DRI for fiber; WWF 20 = partial replacement of WF by WWF ‐providing 20% of the DRI for fiber; CPF 20 = partial replacement of WF by CPF ‐providing 20% of the DRI for fiber; WWF 25 = partial replacement of WF by WWF ‐providing 25% of the DRI for fiber; CPF 25 = partial replacement of WF by CPF ‐providing 25% of the DRI for fiber.

Overall, the models showed a good fit to the experimental data, indicating strong predictive capability.

Of the 18 series (formulation vs. temperature) examined, Page and Logarithmic alternated the lowest RMSE in equal proportions (9/18 each), while Henderson and Pabis (H & P) was not superior in any series (presenting systematically higher RMSE). This pattern is repeated for S, with H & P presenting higher values for this parameter, unlike Page and Logarithmic. As for R^2^_adj_, it remained high (≥0.98) in most cases, however, the Logarithmic model displayed a collapse in CPF 25/45°C (R^2^_adj_ = 0.8991; S = 2.1010; RMSE = 1.7578), whereas, the Page model did not present abrupt breaks of this type throughout the set. As S and RMSE are equivalent measures of residual dispersion, both could confirm the interpretation that Page and Logarithmic deliver the best fits.

**FIGURE 1:**
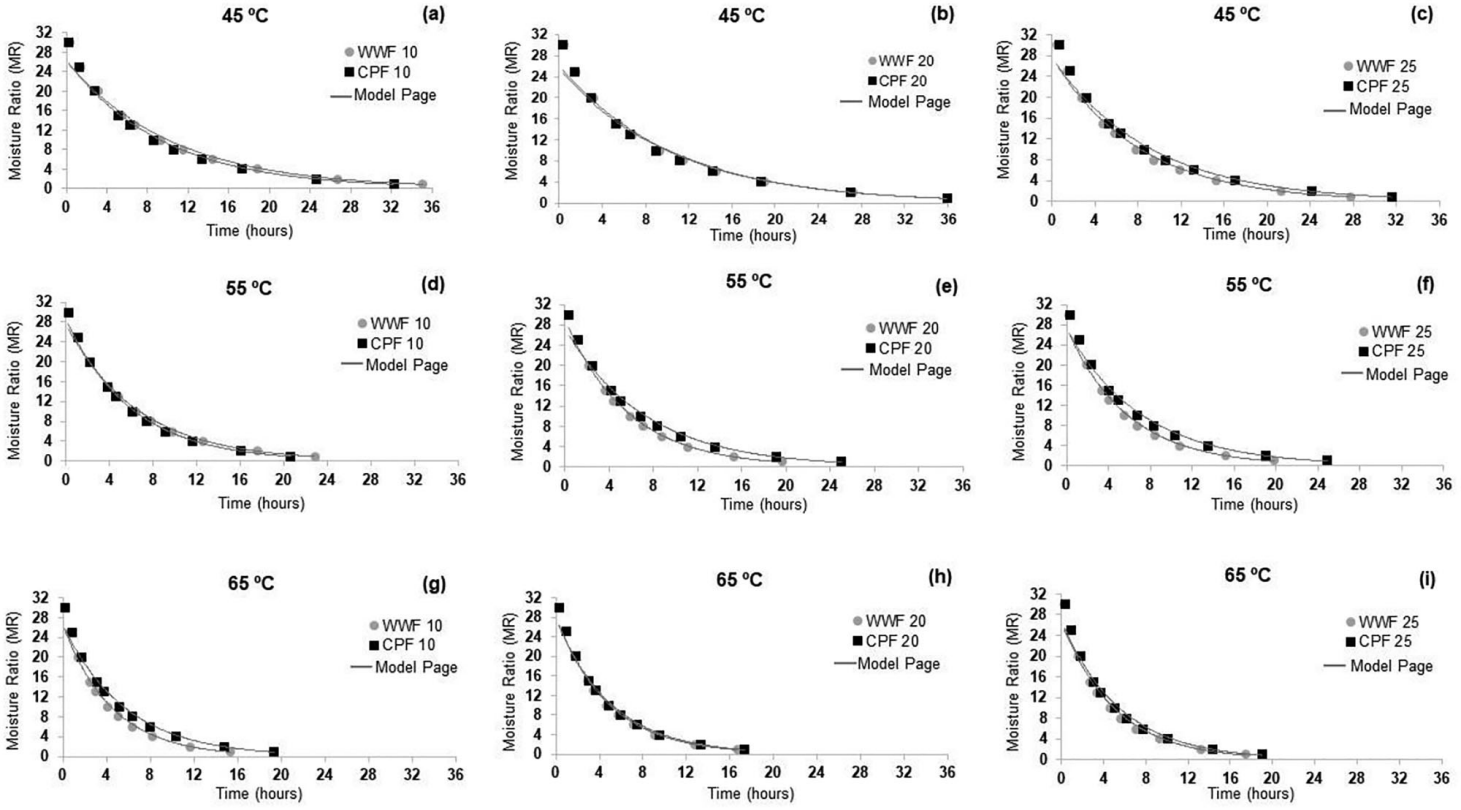
Graphical representation of the drying curves of the pasta samples fitted using the Page model. (a) = drying curve for 10% formulations at 45°C, (b) = drying curve for 20% formulations at 45°C, (c) = drying curve for 25% formulations at 45°C, (d) = drying curve for 10% formulations at 55°C, (e) = drying curve for 20% formulations at 55°C, (f) = drying curve for 25% formulations at 55°C, (g) = drying curve for 10% formulations at 65°C, (h) = drying curve for 20% formulations at 65°C, and (i) = drying curve for 25% formulations at 65°C. WWF 10 = partial replacement of WF by WWF providing ‐10% of the DRI for fiber; CPF 10 = partial replacement of WF by CPF ‐providing 10% of the DRI for fiber; WWF 20 = partial replacement of WF by WWF ‐providing 20% of the DRI for fiber; CPF 20 = partial replacement of WF by CPF ‐providing 20% of the DRI for fiber; WWF 25 = partial replacement of WF by WWF ‐providing 25% of the DRI for fiber; CPF 25 = partial replacement of WF by CPF ‐providing 25% of the DRI for fiber.

Effectiveness was demonstrated by both models—Page and Logarithmic—considering the higher R^2^_adj_ consistently and lower S/RMSE. A practical advantage of the Page model, due to robustness in its series and the support of studies in the area, is successful application for food matrices such as fruits, vegetables and cereals, frequently surpassing other mathematical models due to the good relationship between precision and simplicity, which is consistent with the balance observed in the data obtained in this study (Onwude et al. 2016; Getahun et al. 2021). Thus, it was decided to use Page for fitting the drying curves, allowing the visualization of the influence of temperature and replacement level on the moisture ratio loss rate during drying time (Figure 1), highlighting Logarithmic as a possible secondary comparator model.

Analyzing the graphs, it is evident that temperature had a strong influence on the drying behavior of pasta. Increasing the temperature from 45°C to 65°C led to a reduction of nearly half the time required to reach low moisture content (± 8%), as shown by the sharper declines in the drying curves at 65°C (Figures g, h, and i). This effect can be explained by the higher supply of thermal energy, which facilitates the evaporation of free water present in the food matrix (Tolve et al. 2020).

Pastas formulated with CPF exhibited slower drying at intermediate temperatures (55°C), especially for those with higher substitution levels (20% and 25%) (Figures e and f), resulting in drying times approximately 4 h longer than those formulated with WWF. However, when the temperature increased to 65°C, this difference was reduced to around 1 h (Figures h and i). This behavior may be attributed to the higher content of insoluble fibers, greater water absorption capacity, and potentially increased porosity of the CPF dough matrix, which hinder moisture removal (Tolve et al. 2020).

Drying at 65°C proved to be more efficient for all formulations, as evidenced by the rapid drop in moisture content during the initial hours of the process (Figures g, h, and i). This is likely due to the high temperature, promoting faster evaporation of free water. D'Amico et al. (2015) evaluated the application of high temperatures (60°C, 80°C, and 100°C) in the drying of gluten‐free pasta and found that they improved texture properties, such as elasticity and cooking behavior.

Moreover, the impact of increased fiber substitution was less pronounced at 65°C. Although the pasta formulations with 25% fiber still required more time to dry compared to the others, the difference was less significant at the higher temperature than at 45°C or 55°C. Therefore, drying at 65°C appears to have partially overcome the physical barrier imposed by the increased fiber content.

### 3.3 Optimal Cooking Time (OCT)

The OCT is an operational process marker that indicates the technological cooking point of pasta products; this parameter reflects mainly the effective hydration of the matrix and the advancement of starch gelatinization (Bresciani et al. 2022). The OCT of the developed pasta was evaluated to verify possible changes in this parameter in relation to the drying temperatures (Table 4).

**TABLE 4:** Results of the OCT analysis, in minutes, for dried pasta at each tested drying temperature.

	OCT (min.)					
Temperature (°C)	WWF 10	CPF 10	WWF 20	CPF 20	WWF 25	CPF 25
**45**	9.15 ± 0.07^a^	9.10 ± 0.14^a^	9.30 ± 0.35^a^	9.40 ± 0.14^a^	11.3 ± 0.07^b^	10.3 ± 0.14^c^
**55**	8.75 ± 0.35^a^	8.20 ± 0.14^a^	9.32 ± 0.11^ab^	9.03 ± 0.04^ab^	11.3 ± 0.10^c^	10.2 ± 0.04^d^
**65**	8.75 ± 0.35^a^	9.05 ± 0.07^a^	9.35 ± 0.07^a^	9.40 ± 0.14^a^	10.1 ± 0.14^b^	10.0 ± 0.04^ab^

The results are expressed as mean ± standard deviation. Means followed by the same letter in the same row do not differ significantly according to ANOVA followed by Tukey's test (*p* < 0.05). OCT = Optimal Cooking Time. WWF 10 = partial replacement of WF by WWF providing ‐10% of the DRI for fiber; CPF 10 = partial replacement of WF by CPF ‐providing 10% of the DRI for fiber; WWF 20 = partial replacement of WF by WWF ‐providing 20% of the DRI for fiber; CPF 20 = partial replacement of WF by CPF ‐providing 20% of the DRI for fiber; WWF 25 = partial replacement of WF by WWF ‐providing 25% of the DRI for fiber; CPF 25 = partial replacement of WF by CPF ‐providing 25% of the DRI for fiber.

In Table 4, when increasing the substitution level to 25% (WWF 25/CPF 25), an increase in OCT was observed for the temperatures of 45°C–55°C, which may be related to a delay in starch gelatinization, probably influenced by the higher presence of fibers in the matrix (Ermişer and Yalçın 2021). However, at 65°C, these differences are minimized, resulting in OCTs that are very close between the formulations, suggesting that the drying process with the higher temperature may have resulted in a matrix more efficient with respect to water absorption during cooking, thus reducing the time to reach the ideal technological point.

In this study, the OCT was used as a process metric (point of effective hydration of the pasta). A higher OCT proportional to the increase in fibers of the samples was observed; this behavior is consistent with what is observed for fiber‐enriched pasta, which can result in a lower hydration rate and a delay in gelatinization, prolonging the cooking time (Ermişer and Yalçın 2021).

According to the literature, processes that decrease the OCT are more effective, highlighting that the temperature of 65°C compared to milder temperatures may be more effective. Ermişer and Yalçın (2021), when investigating the cooking properties of spaghetti enriched with dehulled barley flour, found OCTs ranging from 13–15 min, being higher than those observed in this study.

Faster cooking tends to preserve desirable characteristics of pasta, avoiding excessive hydration and, consequently, possible quality losses. The reduction of cooking time, combined with the maintenance of quality, can be advantageous for the consumer who seeks greater practicality in daily life, in addition to contributing to the commercial viability of fiber‐enriched products (Bresciani et al. 2022).

Given this, the temperature of 65°C was adopted for the drying process of the pasta, justified by the combination between process speed (Figure 1) and shorter cooking time.

### 3.4 Physicochemical Composition of the Selected Dried Pasta

For the physicochemical composition outcomes, the samples were characterized at 65°C. This choice aimed to isolate the effect of the flours (CPF vs. WWF) without temperature as a parameter, since the drying kinetics were carried out with the objective of defining the ideal temperature for the pasta production process while preserving product integrity. According to the data, the temperature of 65°C offered the best process performance, presenting a high fit of the models, allied to shorter drying time and shorter OCT. Moreover, by fixing a single drying temperature, analytical comparability between formulations could be ensured, and sources of variability in the extraction and/or quantification of bioactive compounds are avoided, allowing a direct and clean comparison between flours under a single and standardized condition.

Moisture content was analyzed, as it influences the shelf life; ash content, which indicates the presence of minerals that can affect technological properties; and acidity, which reflects product quality, since the presence of free acids in the pasta can compromise the product's integrity and microbiological safety during storage (Yang et al. 2022). Furthermore, these determinations are part of the analyses required by Brazilian legislation as identity and quality parameters for dried pasta (RDC n°. 93, October 31, 2000) (Brazil 2000). The results are presented in Table 5.

**TABLE 5:** Results of physicochemical composition of dried pasta at 65°C.

Parameters (%)	WWF 10	CPF 10	WWF 20	CPF 20	WWF 25	CPF 25
**Moisture (%)**	7.30 ± 0.20^a^	9.00 ± 0.20^b^	7.70 ± 0.40^a^	8.50 ± 0.50^ab^	8.00 ± 0.20^a^	8.80 ± 0.20^b^
**Ash (%)**	1.04 ± 0.01^a^	1.19 ± 0.03^a^	1.24 ± 0.04^a^	1.52 ± 0.03^b^	1.36 ± 0.03^ab^	1.84 ± 0.03^c^
**Acidez (% v/m)**	0.60 ± 0.04^a^	0.77 ± 0.06^b^	0.77 ± 0.06^b^	1.17 ± 0.08^c^	0.85 ± 0.06^b^	1.54 ± 0.08^d^
^1^L*	60.4 ± 0.97^a^	38.3 ± 2.34^b^	52.3 ± 0.53^c^	19.2 ± 2.09^d^	56.3 ± 2.17^ac^	10.9 ± 1.73^e^
^2^a*	5.30 ± 0.75^a^	19.4 ± 0.72^b^	7.70 ± 0.30^c^	30.3 ± 0.76^d^	7.87 ± 0.42^c^	33.8 ± 1.41^e^
^3^b*	42.3 ± 2.40^a^	40.8 ± 1.48^b^	43.6 ± 2.38^c^	30.5 ± 1.45^d^	49.1 ± 2.30^ac^	19.1 ± 2.59^e^

The results are expressed as mean ± standard deviation. Means followed by the same letter in the same row do not differ significantly from each other according to ANOVA followed by Tukey's test (*p* < 0.05). ^1^L* = denotes lightness; ^2^a* = +a indicates red; −a indicates green; ^3^b* = +b indicates yellow; –b indicates blue. WWF 10 = partial replacement of WF by WWF providing ‐10% of the DRI for fiber; CPF 10 = partial replacement of WF by CPF ‐providing 10% of the DRI for fiber; WWF 20 = partial replacement of WF by WWF ‐providing 20% of the DRI for fiber; CPF 20 = partial replacement of WF by CPF ‐providing 20% of the DRI for fiber; WWF 25 = partial replacement of WF by WWF ‐providing 25% of the DRI for fiber; CPF 25 = partial replacement of WF by CPF ‐providing 25% of the DRI for fiber.

Regarding moisture content, it was observed that the pasta samples with CPF showed higher values compared to those with WWF, and this was not affected by the substitution level. The inclusion of CPF may possibly impact the final moisture percentage of the pasta due to its insoluble fiber content and, consequently, the need for adding more water (Table 1). However, the moisture percentages remained within appropriate limits to ensure microbiological stability and safety during storage (< 13%) (Brazil 2000).

Concerning total ash content, the pasta with CPF differed significantly from those with WWF at higher replacement levels (20% and 25%). Nevertheless, the ash content for the highest substitution level is still lower compared to pasta made with a mixture of semolina and hibiscus flour (3.3%) (Bello‐Perez et al. 2024) and expected once this CPF has a higher ash level.

Acidity increased with the fiber substitution level, being higher for pasta with CPF; however, the acidity values are considered safe for this product (Brazil 2000). Yang et al. (2022), when evaluating fresh wheat flour pasta, found that during storage, nutrients (carbohydrates and lipids) were metabolized by microorganisms into organic acids such as citric and lactic acids, resulting in increased product acidity. The authors emphasize the importance of controlling acidity levels concerning microbial growth.

Analyzing the color parameters, the addition of CPF resulted in lower lightness values, with pasta becoming significantly darker as the substitution level increased. This was similarly observed by Makhlouf et al. (2019), who found that increasing the incorporation of whole barley flour in fusilli pasta led to a decrease in L* value compared to the control pasta (from 51 to 42). The opposite trend was noted for the a* parameter, which increased with the substitution level. In their study, Ermişer and Yalçın (2021) reported an increase in a* values from 2.6 in control pasta to 6.7 in pasta enriched with whole and refined barley flour, supporting the findings of this study. A positive a* value indicates a shift towards a redder color in the samples, possibly due to natural pigments such as phenolic compounds, carotenoids, or other antioxidants.

**FIGURE 2:**
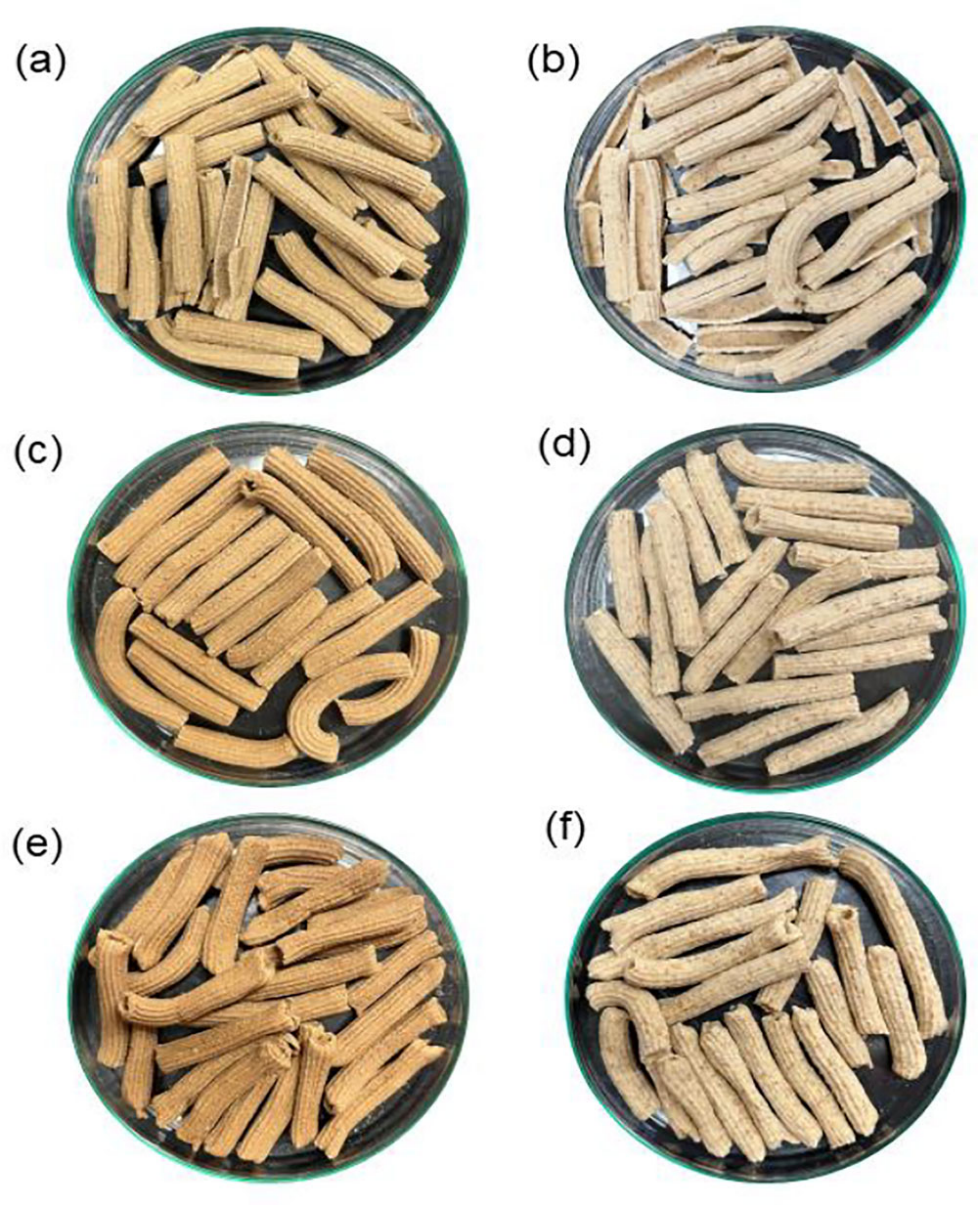
Representative Images of Dried Pasta with different replacement levels produced. (a) = CPF 10: partial replacement of WF by CPF with 10% of the DRI for fibers, (b) = WWF 10: partial replacement of WF by WWF with 10% of the DRI for fibers, (c) = CPF 20: partial replacement of WF by CPF with 20% of the DRI for fibers, (d) = WWF 20: partial replacement of WF by WWF with 20% of DRI for fibers, (e) = CPF 25: partial replacement of WF by CPF with 25% of the DRI for fibers, and (f) = WWF 25: partial replacement of WF by WWF with 25% of the DRI for fibers.

Regarding the b* parameter, an opposite behavior was observed depending on the flour used. While samples with CPF showed decreased b* values, samples with WWF showed increased values with higher substitution levels. According to Makhlouf et al. (2019), higher positive b* values indicate a shift towards yellow coloration, suggesting that samples with WWF exhibited an increase in this color, characteristic of the flour's hue. This behavior was inversely proportional to the a* parameter, indicating that samples with CPF tend to become more reddish as the flour addition increases, while samples with WWF enhance the yellow coloration with increasing flour content. Gerardi et al. (2023) observed a similar behavior when producing pasta enriched with white and red grape flours, where b* values decreased (27 to 3.3) and a* values increased (1.1 to 2.8). It is suggested that the addition of CPF in foods can impart a more intense color, an effect that could be explored in the development of new products. The actual images of the dried pasta samples are shown in Figure 2.

### 3.5 Dietary Fibers and Dry Gluten of the Dried Pasta

Results of dietary fibers, bioactive compound content, and antioxidant potential of the pasta products were presented per 100 g (m/m) of product. Additionally, to facilitate nutritional interpretation, the same data were presented per serving portion of 80 g, an amount widely used as the standard portion for dry pasta, both in the scientific literature and in portion guidelines. These suggest that 80 g is an ideal reference for obtaining the nutritional quality of pasta, rice, and cereals in countries such as Brazil and Italy (Luca et al. 2014; Dello Russo et al. 2021), and for assisting consumers in maintaining a balanced diet and controlling caloric intake based on nutritional needs (Bresciani et al. 2022).

Table 6 presents the results of the analyses of soluble fibers (SF), insoluble fibers (IF), total fibers (TF), and dry gluten at 65°C.

**TABLE 6:** Results of the analysis of dietary fiber and dry gluten per gram in 100 grams and portion (80 g) of dry pasta at 65°C.

Parameters	WWF 10	CPF 10	WWF 20	CPF 20	WWF 25	CPF 25
**Soluble fiber**						
**g/100 g**	0.88 ± 0.09^a^	0.88 ± 0.13^a^	1.22 ± 0.13^b^	0.96 ± 0.07^ab^	1.05 ± 0.35^b^	1.22 ± 0.10^b^
**Portion (80 g)**	0.70 ± 0.07^a^	0.71 ± 0.10^a^	1.00 ± 0.11^b^	0.77 ± 0.06^ab^	0.84 ± 0.28^b^	0.98 ± 0.08^b^
**Insoluble fiber**						
**g/100 g**	3.14 ± 0.26^a^	3.45 ± 0.31^a^	4.99 ± 0.02^b^	5.36 ± 0.18^c^	6.92 ± 0.81^d^	7.40 ± 0.35^e^
**Portion (80 g)**	2.51 ± 0.21^a^	2.76 ± 0.29^a^	4.00 ± 0.01^b^	4.30 ± 0.17^c^	5.54 ± 0.65^d^	5.92 ± 0.28^e^
**^1^Total fiber**						
**g/100 g**	4.02 ± 0.06^a^	4.33 ± 0.18^a^	6.21 ± 0.10^b^	6.36 ± 0.04^b^	7.97 ± 0.09^c^	8.63 ± 0.10^d^
**Portion (80 g)**	3.17 ± 0.04^a^	3.39 ± 0.15^a^	5.00 ± 0.09^b^	5.13 ± 0.02^b^	6.16 ± 0.06^c^	7.05 ± 0.08^d^
**Dry gluten (%)**	7.37 ± 0.05^a^	6.19 ± 0.61^a^	7.56 ± 0.26^ab^	5.93 ± 0.77^ac^	7.83 ± 0.06^ab^	5.83 ± 0.43^ac^

The results are expressed as mean ± standard deviation. Means with the same letter in the same row do not differ significantly from each other according to ANOVA followed by Tukey's test (*p* < 0.05). ^1^Total fibers were calculated as the sum of soluble and insoluble fibers. WWF 10 = partial replacement of WF by WWF providing ‐10% of the DRI for fiber; CPF 10 = partial replacement of WF by CPF ‐providing 10% of the DRI for fiber; WWF 20 = partial replacement of WF by WWF ‐providing 20% of the DRI for fiber; CPF 20 = partial replacement of WF by CPF ‐providing 20% of the DRI for fiber; WWF 25 = partial replacement of WF by WWF ‐providing 25% of the DRI for fiber; CPF 25 = partial replacement of WF by CPF ‐providing 25% of the DRI for fiber.

In general, the developed pasta formulations reached the intended fiber claim levels (10%, 20%, and 25% of the DRI for fiber portion 80 g). Notably, the pastas with the addition of CPF showed fiber contents similar to or higher than those with WWF, despite using considerably less amount of CPF in the formulation (Table 1), highlighting its high efficiency as a functional ingredient for fiber enrichment in products. Consumption of foods rich in dietary fiber can aid in improving intestinal transit, glycemic control, and modulation of the gut microbiota, promoting beneficial health effects (Bello‐Perez et al. 2024).

Regarding soluble fibers, the values observed for the formulations remained similar, with both CPF and WWF enrichment effectively contributing to the soluble fiber content in the pastas. Spaghetti made with barley flour showed higher soluble fiber contents than those found in this study, depending on the substitution level, with values ranging from 2 to 7 g/80 g. However, barley flour contains a higher amount of 9% soluble fiber than CPF and WWF (Ermişer and Yalçın 2021).

For insoluble fibers (IF), it was observed that the pasta formulations containing CPF presented higher values compared to those with WWF, except for the 10% substitution formulations. This difference was expected, given the significantly higher IF content in CPF. However, it is noteworthy that the CPF formulations were prepared with a higher amount of WF and a smaller amount of CPF (Table 1) yet still achieved comparable IF levels.

Bello‐Perez et al. (2024) reported that hibiscus flour contained 49% IF, a value similar to CPF. Consequently, pasta made with 75% semolina and 25% hibiscus flour reached approximately 12.1 g/80 g of IF and 14.1 g/80 g of total fiber, highlighting that flours with a high content of insoluble fiber are effective in increasing the dietary fiber content of food products.

As for dry gluten, a reduction trend was observed in the formulations containing CPF, although the differences were not statistically significant. This behavior was expected, as no gluten was detected in CPF. However, the formulations with CPF included a higher amount of WF compared to those with WWF (Table 1), which helps maintain gluten presence in the pasta. Such a reduction in gluten is common in formulations with alternative ingredients, but it should be monitored to ensure the technological quality of the pasta, particularly in terms of texture and firmness after cooking (Rizzo and Baroni 2018).

Despite the reduction in percentages of dry gluten, the pastas with CPF can possibly offer hydration pathways (capillarity) associated with the fiber fraction and, combined with the drying temperature of 65°C, were able to reach the technological point (OCT) in a time similar to that of the pastas with WWF. In summary, a close OCT could indicate that the point of effective hydration occurred at comparable times under this condition even with a significantly positive (*p* < 0.05) reduction of gluten.

The gradual decrease in gluten content with increasing CPF substitution levels is a relevant technological parameter, since gluten is responsible for forming the protein network that provides structure and elasticity to pasta products (Rizzo and Baroni 2018). Therefore, although the inclusion of CPF offers nutritional benefits, especially through increased fiber content, it is essential to monitor the integrity of the protein matrix to ensure the sensory and technological quality of the final product. Additional studies may be necessary to evaluate the impact of this substitution on the morphological behavior of the dried pasta and on consumer acceptability.

### 3.6 Bioactive Compounds and Antioxidant Potential of the Dried Pastas

The quantitative data of the bioactive compounds of the pasta dried at 65°C are presented in Table 7.

**TABLE 7:** Results of bioactive compounds and antioxidant potential of dried pasta per gram in 100 grams and portion (80 g) of dry at 65°C.

Parameters	WWF 10	CPF 10	WWF 20	CPF 20	WWF 25	CPF 25
**Caffeine**						
**mg/100 g**	nd	50.7 ± 0.72^a^	nd	99.5 ± 0.36^b^	nd	137.2 ± 0.47^c^
**Portion (80 g)**	nd	40.6 ± 0.75^a^	nd	79.6 ± 0.40^b^	nd	109.8 ± 0.49^c^
**Trigonelline**						
**mg/100 g**	*tr*	30.4 ± 0.05^a^	*tr*	53.6 ± 0.11^b^	*tr*	70.6 ± 0.20^c^
**Portion (80 g)**	*tr*	24.3 ± 0.06^a^	*tr*	42.9 ± 0.12^b^	*tr*	56.5 ± 0.23^c^
**5‐CQA**						
**mg/100 g**	nd	6.71 ± 0.02^a^	nd	7.45 ± 0.01^b^	nd	8.92 ± 0.03^c^
**Portion (80 g)**	nd	5.37 ± 0.02^a^	nd	5.96 ± 0.02^b^	nd	7.14 ± 0.04^c^
**Total Phenolic**						
**mg GAE/100 g**	317.3 ± 2.6^a^	330.0 ± 2.7^b^	334.5 ± 2.6^b^	369.0 ± 4.0^c^	344.6 ± 2.6^db^	430.1 ± 7.0^e^
**Portion (80 g)**	251.1 ± 2.1^a^	265.1 ± 2.1^b^	269.5 ± 2.1^b^	298.2 ± 3.2^c^	273.8 ± 2.1^db^	349.8 ± 6.3^e^
**FRAP**						
**µM FeSO^4^/100 g**	1287.7 ± 7.5^a^	1561.1 ± 22.5^b^	1349.2 ± 15.0^c^	1902.9 ± 7.5^d^	1738.7 ± 15.0^e^	2383.7 ± 30.2^f^
**Portion (80 g)**	1030.2 ± 6.0^a^	1248.9 ± 18.0^b^	1079.4 ± 12.0^c^	1522.4 ± 6.0^d^	1390.1 ± 12.0^e^	1906.1 ± 24.2^f^
**DPPH**						
**µM Trolox/100 g**	6.25 ± 0.10^a^	6.27 ± 0.08^a^	6.28 ± 0.07^a^	6.39 ± 0.08^ab^	6.35 ± 0.10^a^	6.54 ± 0.03^c^
**Portion (80 g)**	5.00 ± 0.07^a^	5.01 ± 0.06^a^	5.02 ± 0.05^a^	5.11 ± 0.06^ab^	5.08 ± 0.08^a^	5.23 ± 0.01^c^

The results are expressed as mean ± standard deviation. Means followed by the same letter in the same row do not differ significantly according to ANOVA followed by Tukey's test (*p* < 0.05). GAE = gallic acid equivalents; FRAP = Ferric Reducing Antioxidant Power; DPPH = 2,2‐diphenyl‐1‐picrylhydrazyl. nd = not detected; *tr* = traces, below the quantification limit. WWF 10 = partial replacement of WF by WWF providing ‐10% of the DRI for fiber; CPF 10 = partial replacement of WF by CPF ‐providing 10% of the DRI for fiber; WWF 20 = partial replacement of WF by WWF ‐providing 20% of the DRI for fiber; CPF 20 = partial replacement of WF by CPF ‐providing 20% of the DRI for fiber; WWF 25 = partial replacement of WF by WWF ‐providing 25% of the DRI for fiber; CPF 25 = partial replacement of WF by CPF ‐providing 25% of the DRI for fiber.

Caffeine, trigonelline, and chlorogenic acid (5‐CQA) are the bioactive compounds most associated with coffee and, consequently, with its by‐products (Iriondo‐DeHond et al. 2020). In the pasta formulations with the addition of CPF, a proportional increase in these compounds was observed as the substitution level increased.

Caffeine is widely recognized for its stimulant effects and cognitive benefits, for healthy adults, (EFSA 2015) considers single doses of up to 200 mg as not raising safety concerns. For dry pasta developed, even at the highest level of substitution (CPF 25), caffeine content remained well below these limits. However, caffeine content was not assessed after cooking pasta; given that caffeine is thermally stable but highly water‐soluble, losses during cooking could occur, predominantly by leaching into the cooking water. Literature suggests that cooking noodles in boiling water could promote the leaching of water‐soluble compounds into the cooking medium (Yu et al. 2020). Thus, the values reported for the dry pasta developed are likely overestimates, and future studies should determine the real caffeine content after cooking. In this work caffeine was analyzed as a compositional parameter of the pasta enriched with CPF, since coffee pulp presents in its composition about 0.9%–1.5% caffeine (Tsigkou et al. 2025).

Moreover, according to Wickham and Spriet (2018), there is a growing consumer demand for alternative forms of caffeine intake, beyond the traditional ones of coffee and energy drinks, with the aim of increasing energy, improving concentration, and improving physical performance; in this context, other formats of food products such as cereal bars, chewing gums, and dietary supplements fortified with caffeine have been studied.

Trigonelline is an alkaloid with antioxidant and neuroprotective properties, as is 5‐CQA, a phenolic compound that may help reduce oxidative stress in the body. Therefore, the presence of these compounds in enriched foods may provide health benefits, promoting protection against non‐communicable chronic diseases and improving cognitive function (Wickham and Spriet 2018). Potentially, pasta enriched with CPF emerges as an alternative, offering a practical and diverse way to meet the demand for non‐conventional sources of caffeine intake, in addition to adding functionality (antioxidant) and nutritional benefits, an advantage not observed in pasta formulations containing WWF.

In the study conducted by Tolve et al. (2020), the total phenolic content and antioxidant activity of pasta were positively correlated with the amount of grape pomace added, with 205.6 mg GAE/80 g of total phenolics found in pasta fortified with 10% grape pomace, which is in line with the results found in this study. A significant increase in phenolic content and antioxidant activity measured by the FRAP method was observed in the CPF‐enriched pasta compared to those with WWF. In the study conducted by Aranibar et al. (2018), the authors added partially defatted chia flour to regular wheat pasta. As a result, the fortified pasta showed higher phenolic content, and the antioxidant activity by the FRAP method increased with the concentration of chia flour, compared to the control, like what was observed in this study. In the DPPH assay, no significant differences were observed between formulations, except for CPF 25. These findings are consistent with those of Moreno et al. (2019), who developed savory biscuits enriched with silver skin from coffee, and did not observe significant differences between the control and enriched biscuits in the DPPH assay. However, the total phenolic content nearly doubled. This indicates that the antioxidant capacity of a food product depends on the type of product developed and the specific mechanism of the analytical method used.

## 4 Conclusion

Dried pasta products enriched with coffee pulp flour demonstrated good technological performance, even at higher fiber replacement levels, indicating their commercial viability. The results of this study reinforce the potential of coffee pulp as a functional ingredient for enriching food formulations as an alternative to commercial whole wheat flour, while also contributing to the diversification of raw materials in the food industry. Additionally, the incorporation of coffee pulp into dried pasta increased the total phenolic content and contributed to bioactive and antioxidant compounds, resulting in a functional, appealing product. Partial replacement with up to 25% of the daily recommended intake (DRI) of fiber using coffee pulp flour represents an important strategy to promote increased fiber consumption. It also offers an economical and sustainable use of agro‐industrial residues and enhances the nutritional value of products, aligning with the demand for more sustainable practices and innovation in food product development.

## Author Contributions

**Betsy Gois Santos**: writing – original draft, writing – review and editing, formal analysis, methodology, data curation, conceptualization. **Giovana Toscano Ancillotti**: formal analysis, methodology. **Carolina Hage de Figueiredo**: methodology, formal analysis. **Mariana Aranda Rodrigues**: writing – review and editing. **Vanessa Naciuk Castelo‐branco**: writing – review and editing, conceptualization, supervision. **Thais Matsue Uekane**: writing – review and editing, conceptualization, funding acquisition, supervision.

## Conflicts of Interest

The authors declare no conflicts of interest.

## Supporting information




**Supplementary Materials**: jfds70673‐sup‐0001‐SuppMat.docx

## Data Availability

The authors declare that the data will be made available upon request.
