# Supplementary material for: Study of the Drying Kinetics on the Nutritional and Technological Quality of Dried Pasta Enriched With Coffee Pulp Flour
Source: J Food Sci. 2025 Nov 7;90(11):e70673. doi: 10.1111/1750-3841.70673 (PMC12595288; doi:10.1111/1750-3841.70673)
Supplement: Supplementary file 1 — Supplementary Materials: jfds70673‐sup‐0001‐SuppMat.docx [file JFDS-90-0-s001.docx]

**Supplementary Material**

**Supplementary Material 1.** Parameters of the Page, Logarithmic, and Henderson & Pabis models for the drying curves of the dried pasta 100% White Flour (WF 100%).

| **WF 100%** | | | | | | | | |
| --- | --- | --- | --- | --- | --- | --- | --- | --- |
| **Temperatures (ºC)** | **Model** | **k** | **n** | **a** | **c** | **R²_adj_** | **S** | **RMSE** |
| **45** | Page | 0.010810 | 0.7357 | 36.40 | - | 0.9976 | 0.3233 | 0.2705 |
|  | Logarithmic | 0.005297 | - | 22.73 | 12.920 | 0.9928 | 0.5652 | 0.4729 |
|  | H & P | 0.002373 | - | 34.64 | - | 0.9721 | 1.1100 | 0.9928 |
| **55** | Page | 0.015080 | 0.7318 | 37.18 | - | 0.9565 | 1.736 | 1.4524 |
|  | Logarithmic | 0.006057 | - | 26.67 | 9.337 | 0.9430 | 1.988 | 1.6633 |
|  | H & P | 0.003349 | - | 34.93 | - | 0.9372 | 2.086 | 1.8658 |
| **65** | Page | 0.017280 | 0.7796 | 35.76 | - | 0.9972 | 0.5162 | 0.4319 |
|  | Logarithmic | 0.007745 | - | 29.50 | 5.215 | 0.9913 | 0.9011 | 0.7539 |
|  | H & P | 0.005386 | - | 33.78 | - | 0.9809 | 1.3390 | 1.1976 |

k = drying constant; a, c, and n = model coefficients (dimensionless); R²adj = adjusted coefficient of determination; S = standard error of regression; RMSE **=** Root Mean Squared Error. WF 100% = pasta 100% White Flour.

**Supplementary Material 2.** Graphical representation of the drying curve of the pasta 100% White Flour (WF 100%) fitted using the Page model.

**
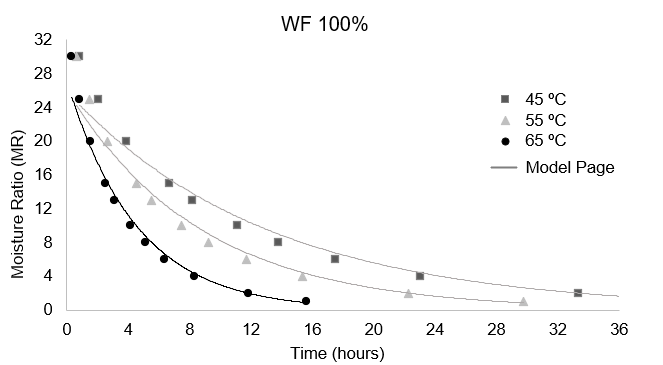
**

Drying curves for 100% white flour formulation at 45, 55 e 65 °C fitted using the Page model.
